# A prospective study of angiogenic markers and postmenopausal breast cancer risk in the prostate, lung, colorectal, and ovarian cancer screening trial

**DOI:** 10.1007/s10552-016-0779-5

**Published:** 2016-06-29

**Authors:** Roni T. Falk, Annetine Cathrine Staff, Gary Bradwin, S. Ananth Karumanchi, Rebecca Troisi

**Affiliations:** Metabolic Epidemiology Branch, Division of Cancer Epidemiology and Genetics, NCI, 9609 Medical Center Drive, Bethesda, MD 20852 USA; Women and Children’s Division, Department of Gynecology and Obstetrics, Oslo University Hospital, Ullevål, 0424 Oslo, Norway; Clinical and Epidemiologic Research Laboratory, Department of Laboratory Medicine, Boston Children’s Hospital, Boston, MA USA; Deparments of Medicine, Obstetrics and Gynecology, Center for Vascular Biology, Beth Israel Deaconess Medical Center, Harvard Medical School, Boston, MA USA; Epidemiology and Biostatistics Program, Division of Cancer Epidemiology and Genetics, National Cancer Institute, 9609 Medical Center Drive, Bethesda, MD 20852 USA

**Keywords:** Breast cancer risk, Angiogenesis, VEGF, sFlt-1, PlGF

## Abstract

**Purpose:**

Pro-angiogenic factors are positively associated with breast tumor staging and poorer prognosis, but their role in the etiology of breast cancer has not been assessed.

**Methods:**

We measured serum levels of the pro-angiogenic vascular endothelial growth factor A (VEGF), and placental growth factor (PlGF) and anti-angiogenic soluble fms-like tyrosine kinase-1 (sFlt-1) in 352 incident breast cancer cases [mean age at diagnosis 67 (range 55–83)] and 352 non-cases in the prostate, lung, colorectal, and ovarian screening trial (women enrolled 1993–2001, followed through 2005) matched on age and date of enrollment. Cases were followed on average 4.2 years from blood draw to diagnosis, range 3.9–12.8 years; 53 % were estrogen receptor positive/progesterone receptor positive (ER+/PR+), and 13 % were ER−/PR−. Quartile-specific hazard ratios (HR) and 95 % confidence intervals (CI) were estimated using weighted Cox proportional hazards regression models adjusted for known breast cancer risk factors. An ordinal variable for the angiogenic markers was used to test for trend in the HR.

**Results:**

Comparing the highest to lowest quartile, multivariable HR were 0.90 for VEGF (95 % CI 0.33–2.43, *p* trend = 0.88), 1.38 for sFlt-1 (95 % CI 0.63–3.04, *p* trend = 0.63), and 0.62 for PlGF (95 % CI 0.19–2.00, *p* trend = 0.73). Risk patterns were not altered when all angiogenic markers were included in the model simultaneously, or by restricting analyses to invasive breast cancers, to cases diagnosed two or more years after blood collection or to ER+ tumors.

**Conclusions:**

There was no evidence of an increased breast cancer risk associated with circulating levels of pro-angiogenic markers VEGF and PlGF or a reduced risk with circulating levels of anti-angiogenic marker sFlt-1.

**Electronic supplementary material:**

The online version of this article (doi:10.1007/s10552-016-0779-5) contains supplementary material, which is available to authorized users.

## Introduction

Numerous studies show poorer survival in women with breast cancer tumors that overexpress angiogenesis-promoting proteins including VEGF (also known as VEGF-A) [1–4] and PlGF (a member of the VEGF family) [5, 6], but the prognostic utility of these markers in newly diagnosed cancer is not clear [1, 7, 8], and their potential etiologic roles in breast cancer have not been well studied. A few studies have compared circulating levels of some of these factors in breast cancer cases to non-cases [9–12], but none have done so in prospectively collected bloods.

While angiogenesis is a quiescent process in most adult tissues, it is critical to normal physiologic processes such as inflammation, wound healing, embryogenesis, and the menstrual cycle, as well as to the pathologic processes of tumor growth and metastasis. It entails a complex coordination between pro- and anti-angiogenic factors, and increasing evidence suggests that sex steroids may regulate their production in a tissue-specific manner. Additionally, women with a history of preeclampsia, who during their pregnancy experience pronounced elevations in the anti-angiogenic factor sFlt-1 (also known as soluble VEGF receptor 1) along with low circulating VEGF and PlGF, have a reduced risk of subsequent breast cancer [13–16]. Elevated levels of sFlt-1 have been noted in uncomplicated pregnancies, although to a lesser degree [16], and some studies suggest altered levels of this marker may persist long after parturition in formerly preeclamptic women [17]. Thus, we speculated that an altered angiogenic profile, which may be induced by hormone-related risk factors and/or dietary and lifestyle choices or persist in women with a history of preeclampsia, may be linked to breast cancer development. Using prospectively collected, pre-diagnostic serum samples from the screening arm of the prostate, lung, colon, and ovary cancer trial (PLCO) [18], we assessed whether healthy women with high levels of VEGF and PlGF along with low circulating sFlt-1 would be at elevated risk of breast cancer.

## Materials and methods

### Study population

Incident breast cancer cases and non-cases were drawn from the 39, 116 female participants, aged 55–74 years, who were randomly assigned from 1993 through 2001, to the screening arm of the multicenter prostate, lung, colorectal, and ovarian cancer screening trial (PLCO) [18]. This study was approved by Institutional Review Boards at the US National Cancer Institute and the 10 participating screening centers. For our study, women were a subset of the participants selected for several previous breast cancer biomarker studies using a stratified case–cohort study design [19], which drew from the population of women in the screening arm who at baseline provided a blood sample; completed the questionnaire and at least one study update; reported no prior history of breast cancer; and provided DNA and gave written informed consent. From this population, we identified 1,141 incident breast cancers diagnosed through 30 June 2005 and matched them to 1,141 non-cases who were alive and free of breast cancer by the end of the follow-up. The non-cases were randomly selected, frequency matched to cases on age at study entry (55–59, 60–64, 65–69, and 70–74 years) and period of blood collection (before or after the median collection date, 30 September 1997). Of these 2,282 women, 424 (37.1 %) breast cancer cases and 506 (44.3 %) non-cases were postmenopausal, were not using hormone therapy in the 4 months prior to the baseline collection, did not have a history of bilateral mastectomy, and had no other cancer other than non-melanoma skin cancer diagnosed during the follow-up period. Additional exclusions included the following: insufficient baseline serum (57 cases, 70 non-cases), unreliable values for earlier assays of serum estrogen metabolites (13 cases, 13 non-cases), and two breast cancer cases that could not be histologically confirmed. This left 352 breast cancer cases and 423 non-cases for study. From this group, we selected all 352 cases and 352 non-cases to study angiogenic markers.

### Ascertainment of breast cancer cases

Participants were contacted annually by mail regarding cancer diagnoses occurring within the previous year. Breast cancers obtained from self-reports, next-of-kin, physicians, death certificates, and National Death Index linkage were confirmed by medical records, and tumor characteristics, including histology and hormone receptor status, were abstracted. Only confirmed cases were included in the analysis. Cases were grouped as ductal [International Classification of Diseases for Oncology, 2nd Edition histology code] (8,500), lobular (8,520), and tubular/other/unknown. When quantitative immunohistochemical results were available, tumors were considered estrogen receptor (ER) or progesterone receptor (PR) positive if at least 1 % of cells stained positive [20].

### Serological measurements

Measurement of VEGF, sFlt-1, and PlGF in sera of non-pregnant women is limited, and in preliminary efforts, commercially available assays could not detect PlGF in sera of healthy postmenopausal women. We obtained a more sensitive assay (available for research purposes only) and measured these proteins at the Clinical and Epidemiologic Research Laboratory, Children’s Hospital, Boston, as follows: sFlt-1 was measured via Elecsys chemiluminescent immunoassay (Roche, Germany) [21], and VEGF and PlGF measured by sandwich ELISAs (Quantikine; R&D Systems, Minneapolis MN, USA). The VEGF assay detected the most biologically active isoform VEGF_165_. The limits of detection were 5, 7, and 6 pg/ml for VEGF, PlGF, and sFlt-1, respectively. To monitor the assay reliability, duplicate blinded quality control samples were included in each batch. Within and between batch, CVs were ≤15 % for all markers.

### Statistical methods

Differences between cases and non-cases in baseline characteristics and angiogenic factors were assessed by t tests or Wilcoxon rank sum tests, with VEGF, sFlt-1, and PlGF analyzed on the natural logarithmic scale. To evaluate the association between quartiles of levels of each marker and breast cancer risk, HR and 95 % confidence intervals (CI) were estimated using weighted Cox proportional hazards regression models. Quartile cutpoints of marker concentrations were based on the non-case distribution. Non-cases were weighted by the inverse sample fraction to represent the study cohort; cases were given a weight of 1.0 because no sampling occurred, i.e., all women diagnosed with breast cancer who met the inclusion criteria were selected. Tests for trend were calculated using an ordinal variable for the quartiles. To assess potential confounding, we assessed whether inclusion of known or suspected breast cancer risk factors altered HR from the full model by more than 10 %, using a backwards elimination strategy. All factors meeting this criterion remained in the final model. Variables considered included age at study entry (55–59, 60–64, 65–69, and 70+); family history of breast cancer; personal history of benign breast disease; ages at menarche (<12. 12–13, and 14+), first birth (nulliparous, <20, 20–24, 25–29, and 30+), and menopause (<45, 45–49, 50–54, and 55+); and body mass index (BMI). Sensitivity analyses assessed potential tumor influences on marker levels by excluding women diagnosed with breast cancer within 2 years of blood donation. For women with available pathology information, we evaluated whether HR varied by breast tumor characteristics, including estrogen and progesterone receptor status, histology, and invasive vs in situ behavior. Analyses were performed with SAS Version 9, and proportional hazards ratios were calculated using Proc Surveyphreg [22]. All tests were two-sided, and *p* values <0.05 were considered statistically significant; no adjustment for multiple comparisons was made.

## Results

### Study population characteristics

Table 1 presents the distributions of demographic, medical history, and breast cancer risk factor characteristics of the study participants. Cases and matched non-cases were predominantly Caucasian (87 and 90 % of cases and non-cases, respectively) and were similar with respect to age (by study design), other demographic characteristics, and most medical conditions and reproductive risk factors. However, cases were more likely to have a history of benign breast disease (*p* = 0.045), and of smoking (*p* = 0.008), to have higher BMI at blood draw (*p* = 0.015), and were less likely to report regular aspirin use (*p* = 0.006). Cases were somewhat younger at menarche (*p* = 0.060), and used postmenopausal hormones for a longer duration than non-cases (*p* = 0.066). When the analysis was limited to invasive breast cancers, similar patterns of results were observed.

**Table 1:** Characteristics of participants, PLCO breast cancer study

	Cases		Non-cases		Cohort	*p* value
	*n*	%	*n*	%	Wt%	
	352		352			
Age (year) at study entry						
55–59	93	26.4	93	26.4	30.3	
60–64	105	29.8	105	29.8	28.4	
65–69	93	26.4	93	26.4	23.2	
70+	61	17.3	61	17.3	18.1	
Race						
White, non-Hispanic	307	87.2	316	89.8	89.8	
Black, non-Hispanic	27	7.7	12	3.4	3.6	
Hispanic	4	1.1	5	1.4	1.4	
Asian/Pacific Islander/American Indian	14	4.0	19	5.4	5.1	
Education						
≤High school (HS)	128	36.4	133	37.8	37.9	
Post-HS/some college	125	35.5	122	34.7	34.5	
College	46	13.1	49	13.9	13.8	
Postgraduate	53	15.1	48	13.6	13.8	
Reproductive risk factors						
Age (year) at menarche						
<12	76	21.6	51	14.5	14.0	
12–13	185	52.6	196	55.7	57.0	
14+	91	25.9	103	29.3	29.0	0.06
Age (year) at menopause						
≤45	81	23.0	67	19.0	18.8	
45–49	88	25.0	100	28.4	28.1	
50–54	143	40.6	134	38.1	38.5	
55+	37	10.5	49	13.9	14.6	0.38
Type of menopause						
Natural	268	76.1	272	77.3	76.5	
Bilateral oophorectomy	17	4.8	17	4.8	5.4	
Hysterectomy alone	57	16.2	51	13.5	14.5	
Other	10	2.8	12	3.4	3.6	0.36
Years oral contraceptive use						
None/unk	193	54.8	192	54.6	53.2	
<1	55	15.6	46	13.1	13.3	
2–5	60	17.0	58	16.5	17.2	
6–9	18	5.1	30	8.5	8.8	
10+	26	7.4	26	7.4	7.4	0.50
Years menopausal hormone use						
None/unk	240	68.2	216	61.4	61.4	
<1	62	17.6	65	18.5	18.8	
2–5	31	8.8	48	13.7	13.0	
6–9	5	1.4	14	4.0	4.1	
10+	14	4.0	9	2.6	2.6	0.07
Parity						
Nulliparous	30	8.8	33	9.3	8.5	
1–2	86	24.4	60	17.0	17.0	
3+	235	66.8	262	74.4	74.4	0.11
Age (year) at first birth						
<20	64	18.2	86	24.4	24.7	
20–24	158	44.9	156	44.3	44.6	
25–29	69	19.6	62	17.6	17.1	
30+	30	8.5	18	5.1	4.7	0.28
Other breast cancer risk factors						
Family history breast cancer	69	19.6	59	16.8	17.3	0.59
History of benign breast disease	94	27.1	71	23.8	20.2	0.05
BMI at blood draw (kg/m^2^)						
18 to <25	102	29.0	137	38.9	37.5	
25 to <30	151	42.9	124	34.4	34.7	
30+	99	28.1	91	25.9	26.8	0.02
Smoking history						
Ever	165	46.9	130	36.9	37.3	
Current	30	8.5	31	8.8	8.8	
Former	135	38.4	99	28.1	28.4	
Never	187	53.1	222	63.1	62.7	0.01
Medical conditions						
Tubal ligation	57	16.2	74	21.0	21.1	0.10
Benign ovarian tumor/cyst	39	11.1	34	9.7	9.4	0.41
Uterine fibroid tumors	52	14.8	51	14.5	14.3	0.76
Endometriosis	25	7.1	19	5.4	5.7	0.30
Family history any cancer	229	65.1	216	61.4	60.4	0.31
Hypertensive	125	35.5	111	31.5	31.3	0.62
Myocardial infarction	21	6.0	16	4.5	4.5	0.41
Stroke	13	3.7	10	2.8	2.7	0.53
Diverticulitis	32	9.1	36	10.2	9.8	0.61
Diabetes	27	7.7	22	6.3	6.3	0.45
Gallstones/inflammation	49	13.9	46	13.1	13.2	0.78
Arthritis	154	43.8	155	44.0	43.4	0.94
Osteoporosis	31	8.8	36	10.2	10.6	0.54
Regular aspirin use	122	34.7	158	44.9	45.3	0.01

Most breast tumors were ductal (75.9 %) or lobular (10.5 %) histology and diagnosed with stage 1 or in situ disease (71.9 %). Overall, 52.8 % were ER+/PR+, 13.1 % ER−/PR−, and 8.2 % ER+/PR−; among invasive cancers (*n* = 277), 62.5 % were ER+/PR+ and 15.2 %, ER−/PR− (Table 2).

**Table 2:** Breast cancer tumor characteristics: PLCO breast cancer study

ER / PR status	All		Ductal		Invasive	
	*n*	%	*n*	%	*n*	%
ER+/PR+	186	52.8	136	67.0	173	62.5
ER+/PR-	29	8.2	23	11.3	28	10.1
ER-/PR+	3	0.9	3	1.5	3	1.0
ER-/PR-	46	13.1	41	20.2	42	15.2
NA	88	25.0	63	31.0	31	11.2

	*n*	%
Behavior		
In situ	75	21.3
Invasive	277	78.7
Histology		
Ductal	267	75.9
Lobular	37	10.5
Tubular/other	48	13.6

Stage	*n*	%
0	75	21.3
I	179	50.9
IIA	56	15.9
IIB	30	8.5
IIIA	4	1.1
IIIB	1	0.3
IV	5	1.4
NA	2	0.6

### Angiogenic profile

Overall, weighted, age-adjusted geometric mean levels of PlGF (pg/ml) were similar for cases and non-cases (${\bar{{\text{x}}}}$_cases_ = 19.4, ${\bar{{\text{x}}}}$_non-cases_ = 19.7; *p* = 0.293), VEGF (pg/ml) (${\bar{{\text{x}}}}$_cases_ = 290.8, ${\bar{{\text{x}}}}$_non-cases_ = 288.4; *p* = 0.945), and sFlt-1 (pg/ml) (${\bar{{\text{x}}}}$_cases_ = 88.9, ${\bar{{\text{x}}}}$_non-cases_ = 83.9; *p* = 0.915). No trends in HR were observed for any of the markers (Table 3), and quartile-specific HR were not significant. Including all markers in the model simultaneously did not change the pattern of results (Table 1). Similarly, restricting analysis to cases diagnosed two or more years after blood collection (supplemental Table 1a), to invasive breast cancers (supplemental Table 1b) or to ER+ cancers only (supplemental Table 1c) did not alter the interpretation of findings. HR for all levels of PlGF were nonsignificantly elevated among ER+ cancers, but no trend was evident. Adjustment for circulating estradiol did not alter our findings for VEGF or the other angiogenic markers studied (results not shown).

**Table 3:** Hazard ratios for pro- and anti-angiogenic factors postmenopausal breast cancer, PLCO cohort

	Cases	HR^a^	95 % CI^a^	*p* trend	HR^b^	95 % CI^a^	*p* trend	HR^c^	95 % CI	*p* trend
VEGF pg/ml										
<177	78	1.00	Referent		1.00	Referent		1.00	Referent	
177–304	98	0.60	(0.30, 1.21)		0.62	(0.28, 1.36)		0.63	(0.29, 1.37)	
305–483	83	0.68	(0.30, 1.59)		0.69	(0.28, 1.70)		0.72	(0.29, 1.77)	
484+	93	0.91	(0.38, 2,17)	0.89	0.90	(0.33, 2.43)	0.88	0.90	(0.34, 2.42)	0.91
sFlt-1 pg/ml										
<77.5	115	1.00	Referent		1.00	Referent		1.00	Referent	
77.5–84.4	82	0.64	(0.32, 1.31)		0.72	(0.34, 1.54)		0.73	(0.92, 1.70)	
84.5–90.7	65	0.41	(0.23, 0.73)		0.45	(0.24, 0.83)		0.45	(0.35, 1.52)	
90.8+	90	1.18	(0.57, 2.47)	0.80	1.38	(0.63, 3.04)	0.63	1.45	(0.70, 3.03)	0.61
PlGF pg/ml										
<16.3	78	1.00	Referent		1.00	Referent		1.00	Referent	
16.3–19.0	91	0.90	(0.40, 2.02)		0.72	(0.26, 1.94)		0.71	(0.26, 1.89)	
19.1–21.0	113	1.26	(0.57, 2.79)		1.11	(0.49, 2.53)		1.10	(0.47, 2.56)	
21.1+	70	0.80	(0.31, 2.02)	0.81	0.62	(0.19, 2.0)	0.73	0.60	(0.19, 1.92)	0.7

^a^Adjusted for age at blood draw (55–59, 60–64, 65–69, and 70–74)

^b^Adjusted for age at blood draw (55–59, 60–64, 65–69, and 70–74), history of benign breast disease, family history of breast cancer, age at menarche (<12, 12–13, 14+), age at first live birth (nulliparous, <20, 20–24, 25–29, 30+) smoking history (current, former, and never), and BMI at blood draw (continuous)

^c^Models include all of the angiogenic factors as well as the variables above

**Fig. 1:**
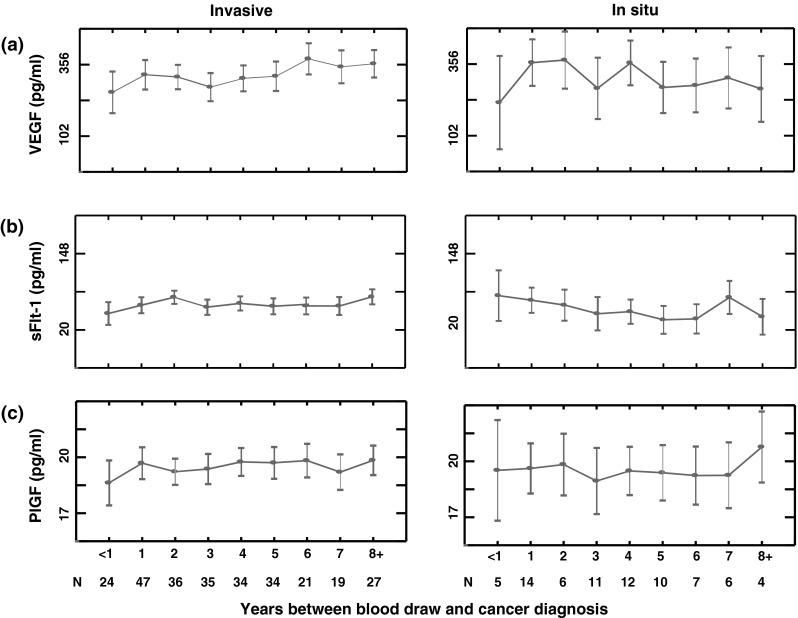
Age-adjusted mean angiogenic factor concentrations by years between blood draw and diagnosis among invasive and in situ breast cancer cases (VEGF, sFlt-1, and PlGF, **a**–**c**, respectively)

Among women with invasive cancer, VEGF levels were lowest in those diagnosed within the first year after blood collection, and values tended to increase nonsignificantly the longer the time between blood draw and diagnosis (Fig. 1a, *p* = 0.099). On average, levels in those diagnosed within the first year after blood donation were 250 pg/ml; this increased to 350 pg/ml in women diagnosed eight or more years after blood donation. sFlt-1 and PlGF did not vary consistently by recency of blood collection (Fig. 1b, c). Among non-cases, levels of all the markers were similar across the age groupings (supplemental Fig. 1a–c).

## Discussion

This first study to evaluate pre-diagnostic serum levels of pro- and anti-angiogenic factors did not demonstrate a link between these biomarkers and postmenopausal breast cancer risk. Circulating angiogenic markers have been used for breast cancer staging and prognosis [3], but efforts to study their role as etiologic agents or early diagnostic markers of breast cancer are limited, and to our knowledge, no epidemiologic study has assessed risks using samples collected several years prior to postmenopausal breast cancer diagnosis. In studies comparing marker levels in newly diagnosed cases to non-cases, most [9, 11, 12], but not all [10, 23–25], found higher levels of circulating VEGF or PlGF in breast cancer cases than in non-cases, which may reflect local tumor production [26]. Contrary to this, we found VEGF was lowest in women diagnosed with invasive breast cancer close to the time of blood donation (within the year of enrollment in the cohort), and unexpectedly, levels tended to be higher among women diagnosed several years after blood donation. For the relatively small number of women with DCIS (*n* = 75), no discernible pattern was observed between VEGF and recency of blood donation, and overall, VEGF levels were comparable among women with invasive and in situ disease. Since the majority of women with invasive cancer were diagnosed with stage 1 disease, our study does not support a role for serum VEGF as a marker of early progression from DCIS to invasive breast cancer. No studies have evaluated a similar role for sFlt-1 and PlGF in breast cancer etiology, in part because previous assays have not been sensitive enough to detect the low levels found in postmenopausal or non-pregnant women.

The close physiologic relationship between endocrine function and angiogenesis, and suggestions that sex steroids regulate the balance of angiogenic factors, particularly VEGF, in a tissue-specific manner [3, 27], provide some support for angiogenic imbalance in breast cancer development. In healthy premenopausal women, VEGF levels in breast tissue are high during the luteal phase of the menstrual cycle when neovascularization occurs and both progesterone and estradiol levels are high [28, 29]. Circulating VEGF is elevated during ovulation [30], in women using exogenous hormones [31], and in those undergoing IVF [32]. We did not observe strong correlations between circulating estradiol and VEGF, and adjustment for this did not alter our findings for any of the angiogenic markers. Finally, recent investigations have linked circulating angiogenic factors to several breast cancer risk factors thought to operate at least in part by hormonal mechanisms, including physical exercise and postmenopausal obesity. Exercise causes a transient but significant increase in circulating levels of sFlt-1 and decrease in VEGF [33], while overweight and obese women have higher VEGF levels than normal weight women [34].

Why pregnancies complicated by preeclampsia attenuate maternal breast cancer risk later in life is not fully understood [14, 15], but may involve changes in mammographic density [35] or circulating sex steroids and/or growth factors [36], or reflect underlying biologic characteristics that are associated with both pregnancy complications and reduced breast cancer risk [17]. We could not assess whether women with a preeclamptic pregnancy experienced a reduced breast cancer risk in this population since this information was not available, although given the rarity of the condition (5–8 % of pregnancies), the number of women in the PLCO cohort with such a history should be low. Similar to our findings, the one study to measure serum PlGF and sFlt-1 during pregnancy did not link these markers to subsequent breast cancer risk [23], although the follow-up time was short (10 years after the index pregnancy) and case accrual was low since most women were still premenopausal.

Reasons for our lack of findings are not clear, but the ongoing debate regarding the clinical utility of circulating VEGF as a prognostic marker in breast cancer may provide some insight [1, 37–39]. Findings across studies are not consistent [40] in part because VEGF has been measured in both serum and plasma, and the absolute values of this marker in these different blood components are very divergent. Since VEGF is sequestered in platelets [39], assay measurements in serum, which is likely contaminated by platelets, are substantially higher than in plasma [41, 42]. While platelet activation has long been recognized in women with breast cancer [37] and higher levels of VEGF are sequestered in platelets of breast cancer cases compared to healthy women [43], it is not known to what extent serum levels of VEGF capture non-tumor vs tumor-derived sources of this marker.

This study had notable strengths, including that the study population was drawn from the PLCO cohort, which provided prospectively collected serum samples, and angiogenic markers were measured using assays available for research purposes that can detect the low values, particularly of PlGF and sFlt-1, found in postmenopausal women. This study also had several weaknesses. The measurement of angiogenic factors occurred at one point in time, which is a common limitation in studies of circulating biomarkers, and the representativeness and stability of these markers over time is not known. We did not have the opportunity to evaluate temporal changes in these markers in individual women; however, our finding of little differences in marker levels in non-cases across the age groups provides some confidence of the stability of these markers in the postmenopause. Additionally, inferences from this study are limited, since only women who were postmenopausal and not using menopausal hormone therapy at the time of blood collection were eligible for study. Finally, as discussed earlier, both the study population and specimen used may not be optimal for assessing the role of angiogenic markers in breast cancer risk, particularly in women who experienced pregnancy complications. Nevertheless, this study does not support the proposition that a pro-angiogenic profile is associated with excess breast cancer risk.

## Electronic supplementary material

Below is the link to the electronic supplementary material.

Box-plots of Angiogenic Factors by Age Group (VEGF, sFlt-1 and PlGF, supplemental figures 1a–1c,  respectively.) (PPTX 43 kb)

Supplementary material 2 (DOCX 16 kb)
