# Supplementary figures and images for: A prospective study of angiogenic markers and postmenopausal breast cancer risk in the prostate, lung, colorectal, and ovarian cancer screening trial
Source: Cancer Causes Control. 2016 Jun 29;27:1009–17. doi: 10.1007/s10552-016-0779-5 (PMC4958123; doi:10.1007/s10552-016-0779-5)

## Slide 1
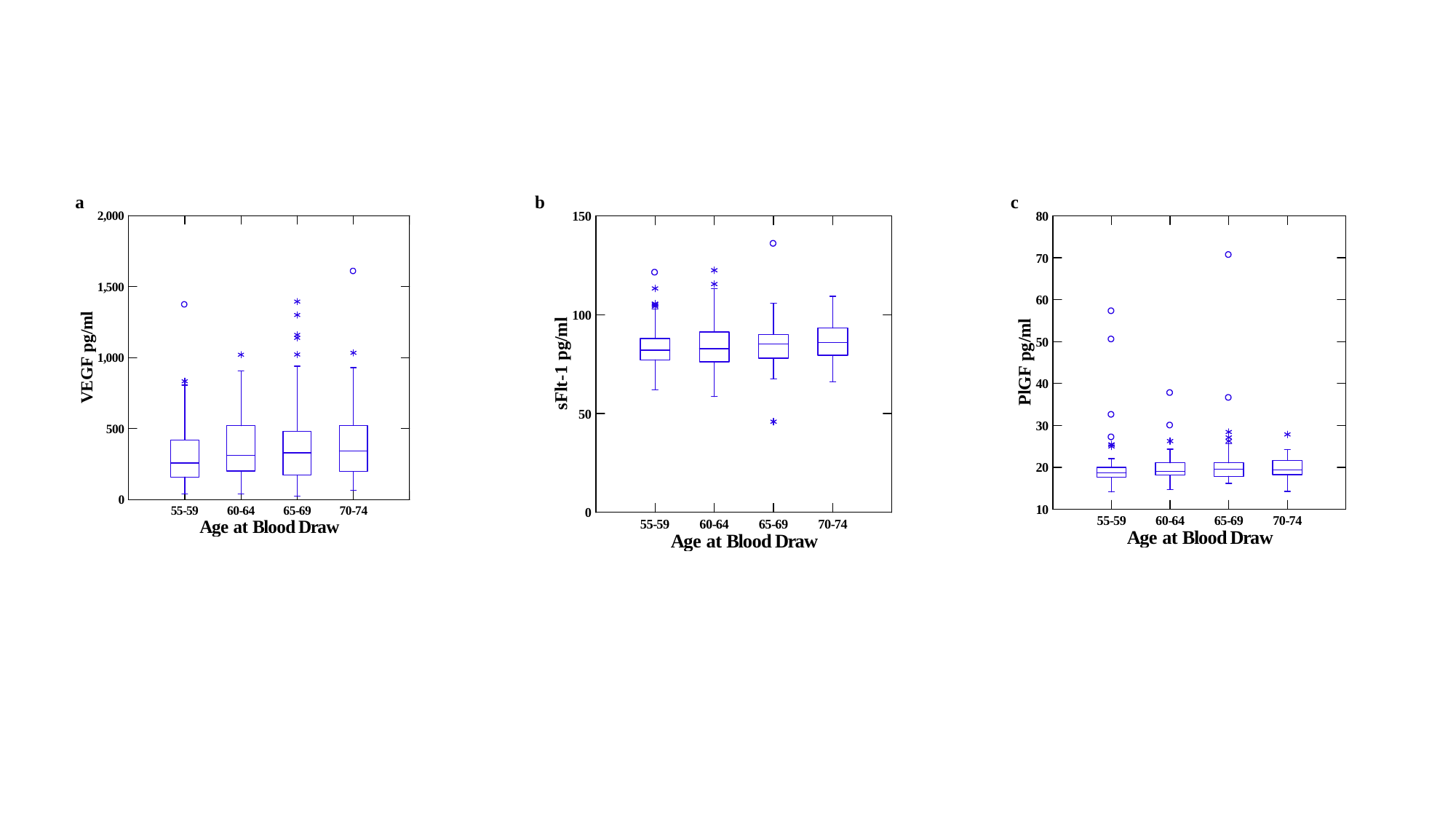

a
b
c

Supplement: Supplementary file 1 — Box-plots of Angiogenic Factors by Age Group (VEGF, sFlt-1 and PlGF, supplemental figures 1a–1c, respectively.) (PPTX 43 kb) [file 10552_2016_779_MOESM1_ESM.pptx]
