# Supplementary material for: A prospective study of angiogenic markers and postmenopausal breast cancer risk in the prostate, lung, colorectal, and ovarian cancer screening trial
Source: Cancer Causes Control. 2016 Jun 29;27:1009–17. doi: 10.1007/s10552-016-0779-5 (PMC4958123; doi:10.1007/s10552-016-0779-5)
Supplement: Supplementary file 2 — Supplementary material 2 (DOCX 16 kb) [file 10552_2016_779_MOESM2_ESM.docx]

| **Supplemental Table 1: Hazard Ratios for Pro- and Anti-angiogenic Factors,**  **Postmenopausal Breast Cancer Study, PLCO cohort** | | | | | |
| --- | --- | --- | --- | --- | --- |
| 1. **Excluding cases diagnosed within 2 years of blood donation** | | | | | |
|  | **Cases** | **HR^1^** | **95% CI** | | **P trend** |
|  | **N** | | | | |
| **VEGF** |  |  |  |  |  |
| **Q1** | 56 | 1.00 | referent | |  |
| **Q2** | 69 | 0.50 | (0.20, 1.25) | |  |
| **Q3** | 64 | 0.66 | (0.23, 1.94) | |  |
| **Q4** | 73 | 0.89 | (0.28, 2.88) | | 0.951 |
|  | | | | | |
| **sFlt-1** |  |  |  | |  |
| **Q1** | 89 | 1.00 | referent | |  |
| **Q2** | 57 | 0.65 | (0.20, 0.78) | |  |
| **Q3** | 49 | 0.40 | (0.58, 3.49) | |  |
| **Q4** | 67 | 1.43 | (0.98, 1.06) | | 0.600 |
|  | | | | | |
| **PlGF** |  |  |  | |  |
| **Q1** | 58 | 1.00 | referent | |  |
| **Q2** | 72 | 0.75 | (0.24, 2.37) | |  |
| **Q3** | 74 | 1.06 | (0.40, 2.82) | |  |
| **Q4** | 58 | 0.71 | (0.18, 2.79) | | 0.771 |
| 1. **Invasive Breast Cancer Only** | | | | |  |
|  | **Cases** | **HR^1^** | | **95% CI** | **P trend** |
| **N** | | | | |  |
| **VEGF** |  |  |  | |  |
| **Q1** | 62 | 1.00 | referent | |  |
| **Q2** | 73 | 0.74 | (0.37, 1.51) | |  |
| **Q3** | 69 | 0.94 | (0.38, 2.35) | |  |
| **Q4** | 73 | 1.13 | (0.41, 3.13) | | 0.701 |
|  |  |  |  | |  |
| **sFlt-1** |  |  |  | |  |
| **Q1** | 88 | 1.00 | referent | |  |
| **Q2** | 61 | 0.80 | (0.33, 1.96) | |  |
| **Q3** | 55 | 0.53 | (0.27, 1.02) | |  |
| **Q4** | 73 | 1.47 | (0.65, 3.32) | | 0.553 |
|  |  |  |  | |  |
| **PlGF** |  |  |  | |  |
| **Q1** | 61 | 1.00 | referent | |  |
| **Q2** | 71 | 1.32 | (0.56, 3.12) | |  |
| **Q3** | 90 | 2.12 | (1.12, 4.02) | |  |
| **Q4** | 55 | 1.48 | (0.58, 3.74) | | 0.229 |
| **Supplemental Table 1, continued** | | | | | |
| **c. ER+ Breast Cancers Only** | | | | | |
|  | **Cases** | **HR^1^** | **95% CI** | | **P trend** |
|  | **N** |  | | | |
| **VEGF** |  | | | | |
| **Q1** | 30 | 1.00 | referent | |  |
| **Q2** | 38 | 0.84 | (0.34, 2.08) | |  |
| **Q3** | 31 | 1.18 | (0.30, 4.58) | |  |
| **Q4** | 34 | 1.12 | (0.24, 5.27) | | 0.805 |
|  | | | | | |
| **sFlt-1** |  |  |  | |  |
| **Q1** | 43 | 1.00 | referent | |  |
| **Q2** | 36 | 0.98 | (0.28, 3.36) | |  |
| **Q3** | 23 | 0.33 | (0.13, 0.83) | |  |
| **Q4** | 31 | 0.85 | (0.24, 2.99) | | 0.539 |
|  | | | | | |
| **PlGF** |  |  |  | |  |
| **Q1** | 23 | 1.00 | referent | |  |
| **Q2** | 31 | 2.15 | (0.55, 8.48) | |  |
| **Q3** | 47 | 3.87 | (1.62, 9.24) | |  |
| **Q4** | 32 | 1.22 | (0.52, 2.87) | | 0.219 |
|  |  |  |  | |  |
| ^1^Adjusted for age (4 categories) , history of benign breast disease, family history of breast cancer, age at menarche (3 categories), age at first live birth (4 categories) smoking history and BMI at blood draw (continuous) | | | | | |
